# Antioxidant, Anti-Inflammatory and Cytotoxic Activity of Phenolic Compound Family Extracted from Raspberries (*Rubus idaeus*): A General Review

**DOI:** 10.3390/antiox11061192

**Published:** 2022-06-17

**Authors:** Alejandra Vanessa Lopez-Corona, Illeen Valencia-Espinosa, Fabio Antonio González-Sánchez, Angélica Lizeth Sánchez-López, Luis Eduardo Garcia-Amezquita, Rebeca Garcia-Varela

**Affiliations:** Escuela de Ingenieria y Ciencias, Tecnologico de Monterrey, Ave. General Ramon Corona 2514, Zapopan 45138, Mexico; a01631208@tec.mx (A.V.L.-C.); a01631150@tec.mx (I.V.-E.); fabiogzz@hotmail.com (F.A.G.-S.); alsl@tec.mx (A.L.S.-L.); garcia.amezquita@tec.mx (L.E.G.-A.)

**Keywords:** raspberry, phenolic compounds, antioxidant activity, anti-inflammatory activity, cytotoxicity

## Abstract

Raspberries (*Rubus idaeus*) possess a wide phenolic family profile; this serves the role of self-protection for the plant. Interest in these compounds have significantly increased, since they have been classified as nutraceuticals due to the positive health effects provided to consumers. Extensive chemical, in vitro and in vivo studies have been performed to prove and validate these benefits and their possible applications as an aid when treating several chronic degenerative diseases, characterized by oxidative stress and an inflammatory response. While many diseases could be co-adjuvanted by the intake of these phenolic compounds, this review will mainly discuss their effects on cancer. Anthocyanins and ellagitannins are known to provide a major antioxidant capacity in raspberries. The aim of this review is to summarize the current knowledge concerning the phenolic compound family of raspberries, and topics discussed include their characterization, biosynthesis, bioavailability, cytotoxicity, antioxidant and anti-inflammatory activities.

## 1. Introduction

Red raspberries (*Rubus idaeus*) are native fruits from Europe. The genera *Rubus* contains approximately 740 species, which are classified into 15 subgenera. Raspberries belong to the subgenus *Idaeobatus* [1]. Raspberries have been studied by the pharmaceutical, cosmetical, agricultural and food industries [2,3,4]. They have become popular and highly distinguished for their flavor and characteristic color. Their red color is produced by a pigment known as anthocyanins, which, in other types of berries, can give a blue or purple pigment [5]. This fruit is known to provide the vitamins, minerals, fatty acids, proteins, carbohydrates and dietary fiber needed for healthy nutrition in humans and animals [1,6]. Additionally, they contain a polyphenolic compound profile associated with the prevention and treatment of some pathologies and chronic degenerative diseases [7]. In plant physiology, a phenolic compound family can act as an antioxidant and antimicrobial agent, and their biosynthesis follows the shikimate and phenylpropanoid pathways, described in the following sections [8,9,10]. While raspberries have been reported to have a wide antioxidant capacity, approximately 75% is due to their anthocyanin and ellagitannin contents and 20% to vitamin C; here, a synergistic effect is produced when in contact with polyphenols; the remaining 5% is attributed to other compounds in the matrix [11,12,13,14].

During the development of several common chronic degenerative diseases, the evidence suggests that reactive oxygen species (ROS) and reactive nitrogen species (RNS) play an important role by being responsible for producing oxidative stress in cells. Oxidative stress occurs when ROS and RNS are present at a higher rate than normal; therefore, the antioxidant defenses cannot cope with this imbalance, and the free radicals are unpaired [15]. Different types of ROS, such as superoxide anion radical (O_2_^−^), ^1^O_2_, hydroxide (HO^−^) or hydrogen peroxide (H_2_O_2_), can be produced in plants when there is an imbalance in the electron transport chain or Calvin reactions [16,17]. Molecules are classified as RNS when they are derivatives from nitric oxide and have a strong oxidizing effect. The most common RNS are nitrosyl cation (NO^+^), nitrosyl anion (NO^−^), nitrogen dioxide (NO_2_), dinitrogen trioxide (N_2_O_3_), peroxynitrite (ONOO^−^), nitrite (NO_2_^−^), nitrate (NO_3_^−^) and nitroxyl (HNO) [18]. There are endogenous and exogenous factors that can increase the level of ROS and RNS production in the body. The endogenous factors are related to the biochemical reactions in enzymatic and nonenzymatic pathways in the cell. On the other hand, exogenous factors are linked to environmental and lifestyle factors, such as pollution, alcohol, drugs, radiation and diet. The excess of free radicals in cells is related to aging and many chronic diseases, such as cancer and diabetes [19,20]. Antioxidant compounds function as potential aids by decreasing oxidative stress when pairing ROS-free electrons [21]. Additionally, chronic diseases are also accompanied by an inflammatory response in most cases. Inflammation is a self-defense response of cells that can be triggered by a pathogen or an injury/damage. The inflammatory response is produced by the immune system and is usually acute. In some diseases, this can become chronic and have a negative effect on the cells. Some of the consequences of chronic inflammation include: damage of macromolecules, DNA, tissues, ROS and RNS accumulation and an increase of oxidative stress [22,23,24]. Most of the anti-inflammatory assays are based on measuring the proinflammatory proteins and associated compounds, such as interleukins, tumor necrosis factor alpha and cytokines, among others [25].

Various phenolic family compounds have been proven to have an antioxidant and anti-inflammatory capacity when administered to humans and animals for epidemiological studies. Therefore, members of the phenolic family are considered nutraceuticals, and this is based on the health benefits they confer. Great interest and resources have been focused in developing sustainable protocols to extract these polyphenols from multiple berries with the purpose of producing supplements with a higher concentration compared to its original source. Nevertheless, side effects have been reported when high doses are consumed, mostly through supplements that have been produced by isolating these compounds from their food matrix [26]. The adverse side effects are liver injuries or stroke. Although some supplements contain multiple bioactive compounds and polyphenols, it could be assumed that the side effects linked to supplements are caused by the phenolic family compounds or, if there is a synergetic effect when combining them, with other vitamins or isolated compounds. These risks may be related to the difficulty in mimicking the in vivo conditions when performing the in vitro models of study [27]. Hence, several authors keep performing in vitro and in vivo assays with high doses of polyphenols to determine their cytotoxicity.

There is a vast quantity of antioxidant, anti-inflammatory and cytotoxicity assays that are the key to performing preclinical and clinical trials before selling these supplements. Multiple studies have shown that phenolic family compounds have the ability to scavenge free radicals [28,29,30,31,32]. These findings are based on the data obtained by applying methodologies to determine their antioxidant activity; such as: the oxygen radical absorbance capacity (ORAC), 2,2-diphenyl-1picrylhydrazyl (DPPH), ferric-reducing antioxidant power (FRAP) and 2,2′-azino-bis(3-ethylbenzothiazoline-6-sulfonic acid (ABTS) assays [33]. The nitric oxygen (NO) assay and protein denaturation inhibition assay are commonly used to evaluate the anti-inflammatory effects on cells. This review discusses the antioxidant and anti-inflammatory activity reported in chemical, in vitro and in vivo assays using phenolic family compounds obtained from raspberries; additionally, it explores the antioxidant and anti-inflammatory activity potential attributed to raspberries due to their phenolic compound contents that act as effective nutraceuticals. Cytotoxicity assays are examined and compared with the reported doses used in cancer cell lines from several published studies. An overview of the metabolism, mechanism of action and the beneficial effects that have been observed in patients with chronic degenerative diseases, such as cancer, when provided with raspberry extracts will be further discussed. Further studies are needed to determine if consuming polyphenols in their original food matrix have increased benefits compared to the isolated forms in the human body.

## 2. Phenolic Compound Family Found in Raspberries

### 2.1. Role of the Phenolic Compound Family in Plant Physiology

Phenolic compounds are the most abundant secondary metabolites in plants, and they play an important physiological role; however, that is not the case in plant development [34]. Bioactive compound profiles may vary depending on conditions such as the growth stage and raspberry variety. Particularly, polyphenols serve a self-protection role in plants, and their concentration can be increased as a response to abiotic and biotic stresses, including pests, bacteria, diseases, ultraviolet light, low temperatures, soil nutrients and drought [35]. Since they are sessile to soil, it is difficult for them to attack pathogens and react against adverse environmental conditions. Plants have developed molecular, physiological and morphological mechanisms to overcome stress, included the production of secondary metabolites [36,37,38]. For example, the synthesis of polyphenols contributes to the stability and robustness of raspberries by adapting certain environmental conditions and as a defense against mechanical damage [10]. Anthocyanins and ellagitannins have been reported as the main contributors of the antioxidant effect in raspberries and other plants from the genera *Rubus* [11,13]. A transcriptomic analysis by RNA-Seq conducted by Gutiérrez-Albanchez et al. showed a plant protection of up to 88% in blackberries (*Rubus ulmifolius*) when the regulating and core genes of the flavonol–anthocyanin pathway were expressed in the plant [39]. Ellagitannins from raspberries were proven to have an in vitro and in situ antifungal capacity against *Geotrichum candidum* [40].

Anthocyanins are mainly found in the vacuoles of plant cells. In raspberries, the ones identified at higher concentrations are: cyanidin-3-*O*-sophoroside, cyanidin-3-*O*-glucosylrutinoside and cyanidin-3-*O*-glucoside [41,42]. Regarding ellagitannins, sanguiin H-6 and lambertianin C have also been found at significant amounts in raspberries [43,44]. In a chemical composition study of raspberry seeds conducted by Kosmala et al., the content of ellagitannins was analyzed. Their results showed that sanguiin H-6 was almost 50% of the total ellagitannins content, whereas lambertianin C represented 34% of the total phenolic content [45]. The reported contents of the phenolic compounds by each author may vary; this is due to the variety in origin, location and the chosen extraction method.

The physicochemical characteristics are linked to biotic and abiotic stresses. Some factors that may contribute to these variations in the content values are harvesting, transportation, storage conditions and applied treatments [46]. Mazur et al. [47] evaluated the raspberry genotype variations in three different harvesting seasons, proving that the phenolic content, color and general characteristics were significantly affected. In a recent study, twelve different cultivars of raspberries from Spain and Morocco were examined to prove that the chemical composition results varied even when applying the same conditions [29]. On the other hand, some authors have focused their studies on a specific phenolic family. Chen et al. evaluated the anthocyanin degradation kinetics on raspberry juices at different times of storage. Their results confirmed that the anthocyanins concentration was reduced when longer storage times were applied [48]. In another study, the ellagitannin and anthocyanin contents were quantified from frozen and fresh raspberries; for both compounds, higher concentrations were found in the frozen samples [49]. Ponder & Hallmann performed a phenolic analysis between conventional and organic cultivars of raspberries; the variables they considered were the type of soil, location, dosage and time of the given fertilizers. The antioxidant activity results showed higher values for raspberries that were cultivated under organic conditions than the conventional method [35]. In brief, the harvesting season, region and storage conditions have a strong effect on the physicochemical properties of raspberries.

### 2.2. Classification

**Figure 1:**
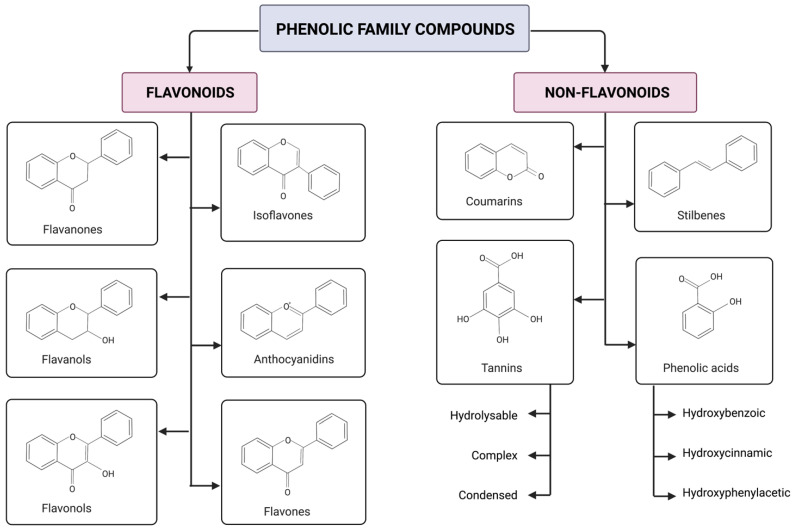
Phenolic family compound basic skeletal structure and their classification in subclasses as stated by [50]. There are two major groups: flavonoids and non-flavonoids. Flavonoids have six subclasses: anthocyanidins, flavonols, flavanones, flavanols, flavones and isoflavones. Anthocyanidins become anthocyanins when sugars are linked in their chemical structures. Non-flavonoids are subdivided into four subclasses: stilbenes, tannins, coumarins and phenolic acids. Tannins are categorized into condensed, hydrolysable and complex. For phenolic acids, a more specified classification is to divide them into three groups: hydroxybenzoic, hydroxyphenylacetic and hydroxycinnamic acids. Created using licensed BioRender (2022).

The phenolic family compounds are divided and classified into two major groups: flavonoids and non-flavonoids, also called simple phenols. Flavonoids have two phenyl rings linked by a pyran ring and may or may not be attached to a sugar molecule. Glycosides are bound to any sugar apart from glucose; on the other hand, glucosides are only bound to glucose, and aglycones are not bound to a sugar molecule. Aglycones have a flavylium ion (2-phenylbenzopyrilium) with a cation function and are composed of two aromatic groups: a benzopyrilium and a phenolic ring [50,51]. As shown in Figure 1, flavonoids are subdivided into six subclasses: anthocyanidins, flavonols, flavanones, flavanols, flavones and isoflavones. Non-flavonoids are subdivided in four subclasses: stilbenes, tannins, coumarins and phenolic acids [51,52]. Phenolic acids are ordered into three categories: hydroxybenzoic, hydroxyphenylacetic and hydroxycinnamic acids [53].

Tannins are categorized in three groups: hydrolysable tannins and condensed tannins; this last one is also known as proanthocyanidins [51]. The characterization is according to the chemical bonds that link their monomers. Hydrolysable tannins have ester bonds, whereas condensed tannins have carbon–carbon and carbon–oxygen–carbon bonds. The conjugations between the condensed and hydrolysable are known as complex tannins [54]. Ellagitannins are categorized as hydrolysable tannins since they release gallic and ellagic acid when hydrolyzed. Gallic acid and multiple hexahydroxydiphenoyl (HHDP) moieties are the core subunits in the ellagitannins chemical structure [55,56]. Anthocyanins are anthocyanidins with sugar molecules coupled to their chemical structure. Anthocyanins and ellagitannins are two of the most representative and studied phenolics found in raspberries [7,47,57].

### 2.3. Phenolic Compound Family Biosynthesis

**Figure 2:**
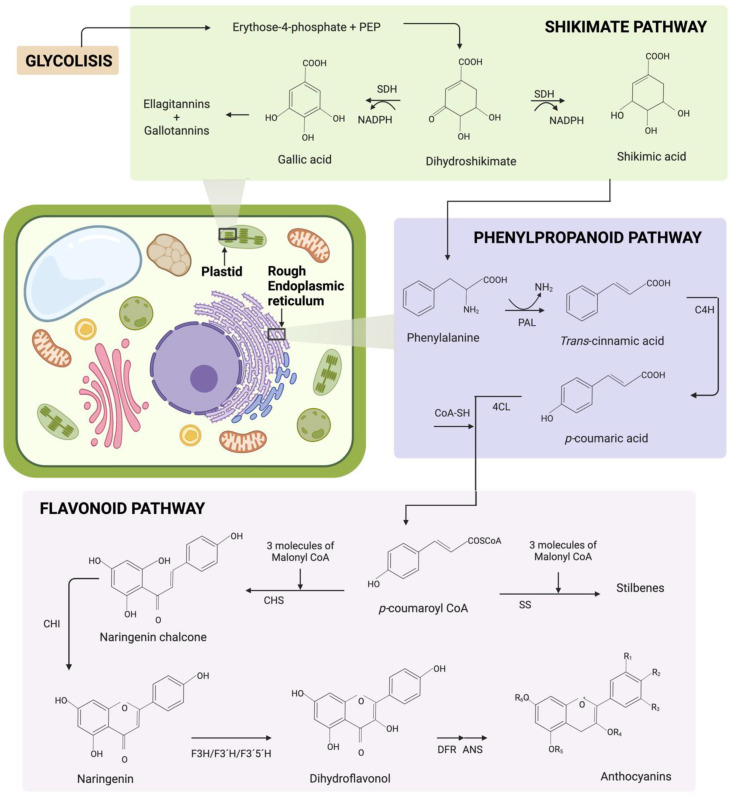
Biosynthesis of the phenolic compound family in the plant cell through the shikimate, phenylpropanoid and flavonoid pathways. The shikimate pathway begins with the reaction of erythrose 4-phosphate and phosphoenolpyruvate (PEP) to produce dehydroshikimate. With this precursor, the enzyme shikimate dehydrogenase (SDH) can catalyze the production of ellagitannins, gallotannins and phenylalanine. The phenylpropanoid pathway begins with the condensation of phenylalanine and acetate by the action of the phenylalanine ammonia lyase enzyme (PAL). Consequently, p-coumaryl CoA is produced by the action of the cinnamate 4-hydoxylase enzyme (C4H) and 4-coumarate CoA ligase (4CL). The precursor of the flavonoid pathway is p-coumaryl CoA, which reacts with three molecules of Malonyl CoA to later produce multiple phenolic family compounds, such as anthocyanins and stilbenes. The enzymes involved in the flavonoid pathway are the following: chalcone synthase (CHS), stilbene synthase (SS), chalcone isomerase (CHI), flavanone 3-hydroxylase (F3H), flavanone 3′-hydroxylase (F3′H), flavanone 3′5′-hydroxylase (F3′5′H), dihydroflavonol 4-reductase (DFR) and anthocyanidin synthase (ANS). Created using licensed BioRender (2022).

Phenolic compound family biosynthesis is known to follow the phenylpropanoid and flavonoid pathways in the rough endoplasmic reticulum of the plant cell. This plant metabolic pathway is based on secondary metabolites generated from the shikimate pathway, which takes place in the plastids [10]. The shikimate pathway synthesizes secondary metabolites such as ellagitannins, gallotannins and phenylalanine, as depicted in Figure 2. The production of secondary metabolites through the shikimate pathway takes place in a wide variety of bacteria, fungi and plants [58]. It starts with the production of dihydroshikimate when erythrose 4-phosphate and phosphoenolpyruvate (PEP) have an aldol condensation reaction [58]. The enzyme shikimate dehydrogenase (SDH) catalyzes the production of gallic and shikimic acid. The final products of this pathway are ellagitannins, gallotannins and phenylalanine [59,60].

The general phenylpropanoid pathway begins with the condensation of phenylalanine and acetate by the action of the phenylalanine ammonia lyase enzyme (PAL) to produce *trans*-cinnamic acid. The cinnamate 4-hydoxylase enzyme (C4H) catalyzes *trans*-cinnamic acid into *p*-coumaric acid. Afterwards, *p*-coumaric acid reacts with 4-coumarate CoA ligase (4CL) to produce *p*-coumaroyl CoA [61]. Overall, in the case of phenolic acids, *p*-coumaric acid functions as a precursor, while flavonoids use *p*-coumaroyl CoA [62]. The flavonoid pathway starts *p*-coumaroyl CoA. Multiple phenolic compound family biosynthesis follows the general phenylpropanoid pathway, as shown in Figure 2, including anthocyanins and stilbenes. For this purpose, enzymes such as chalcone synthase (CHS), stilbene synthase (SS), chalcone isomerase (CHI), flavanone 3-hydroxylase (F3H), flavanone 3′-hydroxylase (F3′H), flavanone 3′5′-hydroxylase (F3′5′H), dihydroflavonol 4-reductase (DFR) and anthocyanidin synthase (ANS) are also required [8,63,64].

## 3. Bioavailability

### 3.1. Anthocyanins

**Figure 3:**
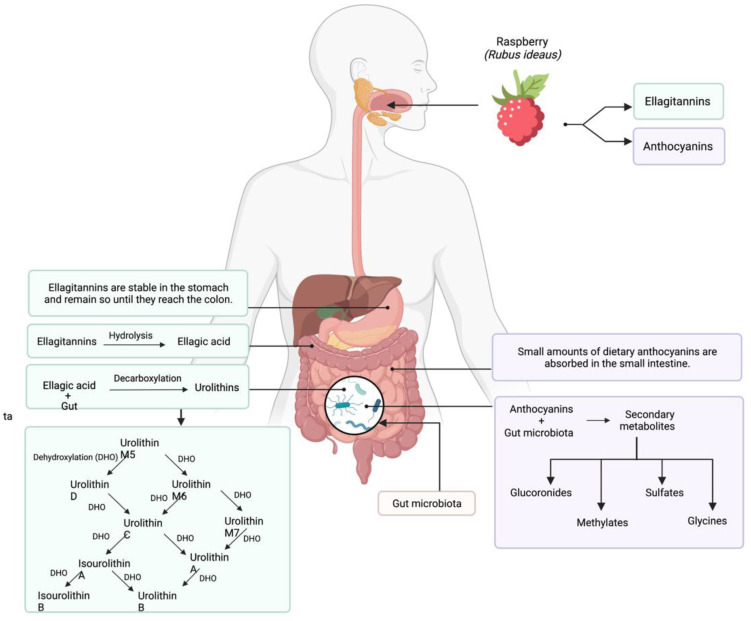
Absorption, metabolism and mechanisms of ellagitannins and anthocyanins in the human body. The gut microbiota plays an important role for their metabolism. First, ellagitannins are transformed into ellagic acid prior to interacting with the gut microbiota. The final metabolites produced in ellagitannin metabolism are urolithins, and their concentrations will vary between individuals. Urolithin-A, isourolithin-A and urolithin-B are the final derivatives from ellagitannins, which can be quantified as plasma and urine. On the other hand, the anthocyanin final metabolites are sulfate, glucuronide, glycine and methyl derivatives. Both ellagitannins and anthocyanins are stable until reaching the small and large intestines, where they will interact with the intestinal microbiota of the host. For anthocyanins, only low amounts do not interact with the gut microbiota, because they are absorbed directly into the small intestine. Abbreviation: dehydroxylation (DHO). Created using licensed BioRender (2022).

Anthocyanins are absorbed and metabolized into the colon; only a low number of dietary anthocyanins are absorbed into the stomach and small intestine when they are in their glycosidic forms [5]. The majority of anthocyanins interact with colon microbiota and other enzymes, and the production of secondary metabolites and catabolic products takes place, as illustrated in Figure 3. The process the gut microbiota employs for anthocyanins metabolism focuses on two steps. First, the separation of aglycones from anthocyanins occurs when their glycosylic links are broken by the actions of the β,D-glucosidase, β,D-glucuronidase and α,L-rhamnosidase enzymes. The second phase takes place when the gut microbiota causes a cleavage of the anthocyanidin heterocycle [65]. Finally, the absorbed anthocyanins are metabolized to sulfate, glycine, glucuronide and methylate derivatives in the intestine epithelium, liver and kidneys [66,67].

Anthocyanin’s metabolism has been a relevant object of study because of their correlative interactions with species found in the gut microbiota. Examining numerous anthocyanins can become complex, since they form a vast subgroup in the flavonoids category. Therefore, multiple authors have focused their research on specific anthocyanins rather than analyzing a wide anthocyanin fraction. Chen et al. employed fresh rat feces to elucidate the metabolism of cyanidin-3-glucoside, cyanidin-3-rutinoside and delphinidin-3-rutinoside; their methodology was mainly based on batch fermentation and HPLC-ESI-MS/MS (High-Performance Liquid Chromatography Electrospray Tandem Mass Spectrometry) analyses. Their findings suggested that the time anthocyanins take to be completely metabolized are between 6 and 8 h. The main metabolites produced are vanillic acid, *p*-coumaric acid, gallic acid, syringic acid, protocatechuic acid and 2,4,6-trihydroxybenzaldehyde [65]. Ludwig et al. conducted an in vivo study with the purpose of evaluating by UHPLC-MS the bioavailability of anthocyanins in the plasma and urine of volunteers fed raspberries. The results in their urine showed that 15% of the excreted intake corresponded to 18 anthocyanin-derived metabolites (methyl, glycine, glucuronide and sulfate derivatives), and 0.007% were anthocyanins. Additionally, they found that cyanidin-3-*O*-glucoside and a cyanidin-*O*-glucuronide were not absorbed when the plasma from volunteers was analyzed [68]. Rodriguez-Mateos et al. performed a metabolomics analysis to quantify the circulating anthocyanin metabolites in the plasma from human volunteers after 2 and 48 h of anthocyanin consumption. In both quantification times, their results showed a total of 63 phenolic metabolites, 3 of them being flavonoid derivatives, and the rest were conjugated and nonconjugated phenolic acid derivatives [69]. A recent study analyzed the urine of volunteers who ingested juices containing anthocyanins. They were able to identify 29 phenolic metabolites; some of them where only identified in a few volunteers’ urine due to the inter-variability in their gut microbiota [70]. Their approach was to evaluate the absorption and excretion of the anthocyanin metabolites. The obtained results suggested that the unabsorbed anthocyanins were excreted in their urine.

### 3.2. Ellagitannins

In the case of ellagitannins, these must be metabolized into urolithins to be absorbed into the human body, as seen in Figure 3 (García-Villalba et al., 2020). The breakdown of ellagitannins into urolithins can vary, because it depends completely on the host microbiota. According to this statement, an interindividual variability could be expected between humans. Individuals can be classified into three different metabotypes of urolithin production: metabotype A produces urolithin-A, and metabotype B produces urolithin-A, isourolithin-A and urolithin-B. Metabotype 0 describes those who do not produce any urolithins [15,56]. A recent study showed that *Gordonibacter urolithinfaciens* and *Gordonibacter pamelaeae,* which were isolated from healthy woman fecal samples, have the capacity to produce urolithins from ellagic acid [71].

Ellagitannins are stable in the stomach environment and remain so until they reach the colon section, where they are metabolized by the intestinal microbiota and enzymes. It has been proven that, under acidic conditions, sanguiin H-6 and lambertianin C are stable, two of the principal ellagitannins found in raspberries [72]. Ellagitannins release ellagic acid, by hydrolysis, when in the colon; this product will later interact with the intestinal microbiota to metabolize into various urolithins derivatives by a decarboxylation of one of the lactone rings in ellagic acid [73,74]. Finally, all types of urolithins are involved in a series of dihydroxylation reactions to produce three final derivates; they all belong to the subgroup of dibenzo-*α*-pyrones, which are known for their biological beneficial effects in the human body [15,75]. Ludwig et al. performed an in vivo study where they fed volunteers raspberries to analyze the bioavailability of ellagitannins, and their results demonstrated that urolithins A and B are excreted almost exclusively as sulfate and glucuronide metabolites, which corresponded to less than 7.0% of the intake [68]. In a recent study, the urine and plasma from human samples were analyzed to identify urolithins after raspberries intake. The urine samples had urolithin-A, isourolithin-A and urolithin-B, whereas the plasma samples had isourolithin-A and urolithin-B [49]. In a previous study, they quantified urolithin-B and urolithin-A in the plasma after six hours of consuming ellagitannin-rich foods [76]. An in vivo study conducted by Piwowarski et al. showed that urolithins A, B and C can reduce intestinal inflammation at a ≥40-μM concentration [77]. Therefore, quantifying the presence of urolithin-A, isourolithin-A and urolithin-B in urine and plasma could be an approach to metabotype identification.

### 3.3. Probiotic and Prebiotic Effect

The published data has focused on the effects that the phenolic compound family have on the gut microbiota, acting as bacterial substrates. A modulatory capacity has been observed on the gut microbiota species concentration when humans consume food and beverages rich in phenolics. The modulated interaction favors health due to the increase in beneficial bacteria species and the decrease in the pathogenic bacteria species [69]. Therefore, phenolics and their metabolites play a prebiotic and probiotic role. Anthocyanin-derived metabolites reduced the concentration of *Bacteroides* and *Clostridium histolyticum*. Additionally, these compounds can increase the population of *Proteobacteria, Fusobacteria, Firmicutes* and *Bacteroidetes* [65]. On the other hand, an increase in the beneficial gut bacteria has been reported when the metabolism of anthocyanins occurs. In a recent study, the fecal concentrations of nine adult men were analyzed to observe if the levels of the bacteria species changed after anthocyanins metabolism. The concentration of the bacteria studied increased after the intervention, and the results were the following: *Bifidobacterium* (5.52 ± 0.88 log_10_ copies/g feces), *Enterococcus* (2.83 ± 0.51 log_10_ copies/g feces) and *Eggerthella lenta* (3.47 ± 1.03 log_10_ copies/g feces) [78]. Recent studies suggest that the consumption of raspberries may increase the population of beneficial bacteria due to their interactions with anthocyanins. Further research to examine which bacteria colonies increase or decrease when being in contact with a specific phenolic compound will provide more information on their probiotic and prebiotic effects. Additionally, an important key point to determine is the phenolic compound dosage in which these effects start.

## 4. Antioxidant Activity

The ROS and RNS are naturally produced during the synthesis of ATP [79]. Oxidative stress occurs when the contents of the ROS and RNS are higher than the antioxidant compounds in the cells, and this state produces an excess of free radicals [11,15]. Negative health effects are associated with the free radical concentration increases at a higher-than-normal rate. This imbalance can be produced either from normal cell metabolisms in situ or from external sources. The ROS and RNS can become carcinogenic agents due to their interference with normal cell growth and function [21]. Additionally, they can oxidize macromolecules such as enzymes, proteins, lipids and DNA. Antioxidants have a high capacity of donating electrons, thus reducing oxidative stress in cells. It has been shown that antioxidant compounds can combat the oxidative stress in cells. Therefore, they have an anticancer effect by affecting different stages of carcinogenesis. A reduction in tumor growth has been associated with diets supplemented with anthocyanins, flavonols, flavonones and hydrolysable tannins [80]. The antioxidant capacity of phenolic compounds, found in raspberries, is greatly due to their radical scavenging activity, since they can easily donate electrons and hydrogen atoms due to their highly conjugated systems and aromatic structures [81]. Compared with ellagitannins, anthocyanins have a higher antioxidant activity, since they can scavenge produced free radicals through two different pathways, whereas ellagitannins only use one pathway. Ellagitannins follow the pathway used by all the hydrolysable tannins, which is characterized by the oxidative coupling of the neighboring galloyl groups [10]. Anthocyanins can couple a free radical by transferring an electron from their hydroxyl groups found in their B-ring and/or from the oxonium ion on the anthocyanin C-ring [31]. In the research conducted by Dobani et al., ex vivo fermentation studies with raspberries were performed to evaluate the antioxidant effect from their phenolic catabolites; the results showed a significant modulation in the Nrf2-ARE pathway, which is involved in oxidative stress, and, therefore, decreased the production of ROS and RNS [82].

There have been several methodologies reported to evaluate the chemical antioxidant capacity of the phenolic compound family. The most used are the ABTS, DPPH, FRAP and ORAC assays. Furthermore, they are divided into two groups: a hydrogen atom transfer-based assay and electron transfer-based assays [33]. These assays have been applied in multiple experiments to extracts from different sources. All the results are quantified using a spectrophotometer and compared with a calibration curve made specifically for each assay. The published research commonly performed at least two assays to gain more accurate results; since they each have different principles, different antioxidant capacity results can be obtained.

Extensive studies have reported the results from applying several of the previously mentioned assays on a raspberry matrix [83]. It is worth mentioning that the type of solvent, incubation times and pretreatments applied to the extract can influence the variations in the antioxidant activity results [84]. As shown in Table 1, the antioxidant capacity for raspberries has been evaluated through chemical, in vitro and in vivo assays. The units in which the results are reported vary according to the standard used by each author. Additionally, another way to report results is percentage inhibition using the following equation (Equation (1)):

(1) $$\mathrm{Inhibition}\;\%=\left(\frac{\mathrm{initial}\;\mathrm{absorbance}-\mathrm{final}\;\mathrm{absorbance}}{\mathrm{initial}\;\mathrm{absorbance}}\right)\times 100$$

The variations between the results when using different antioxidant capacity assays (Table 1) is due to the affinity some compounds have to the reagents. How phenolic compounds couple a free radical by transferring an electron varies between them. Antioxidants are categorized into lipophilic and hydrophilic, corresponding to their solubility properties. Raspberries have both lipophilic and hydrophilic fractions that can be isolated with solvents and different techniques, such as ultrasound and high-pressure extraction processes. As reported by Kryževičiute et al., supercritical fluid extraction with carbon dioxide and pressurized liquid extraction are recommended technologies to obtain high yields of both lipophilic and hydrophilic fractions from raspberries [85]. Prior to a more sophisticated extraction, an ultrasound is generally used to break the fruit tissue to favor a more efficient matter transference for further separation. The ABTS, FRAP, DPPH and ORAC can quantify both lipophilic and hydrophilic antioxidants from a sample [86].

**Table 1:** Chemical, in vitro and in vivo antioxidant capacity assays performed on raspberries (*Rubusidaeus*).

Source	Assays	Results	Assessed Compounds	Reference
Raspberry extracts	Chemical: ABTS, FRAP and DPPH	Higher antioxidant capacity when using ABTS and FRAP assays compared to DPPH assay. Average results by means of the FRAP assay were 20% higher compared to ABTS assay and 35% higher compared to DPPH assay. Results are expressed as µmol Trolox/g fresh weight. DPPH (507–850), FRAP (743–1083) and ABTS (679–1003). Samples concentrations were 0.25 g/mL.	Total phenolics	[29]
Blended raspberry extracts	Chemical: ABTS, FRAP and DPPH	The highest antioxidant capacity results were obtained with the FRAP assay, and the lowest with DPPH. All antioxidant activities were expressed as mg of ascorbic acid equivalent (AAE)/g of the sample; DPPH = 1.63 ± 0.02, ABTS = 1.83 ± 0.05, FRAP = 2.32 ± 0.09. Average results by means of the FRAP assay were 27% higher compared to ABTS assay and 42% higher compared to DPPH assay.	Total phenols, total flavonoids, gallic acid, caffeic acid, chlorogenic acid, caftaric acid, ferulic acid, syringic acid, protocatechuic acid, epicatechin, quercetin-3-glucoronide, quercetin-3-glucoside and kaempferol-3-glucoside.	[87]
Raspberry extracts	Chemical: ABTS and DPPH	Average results by means of the DPPH assay were 25% lower compared to ABTS assay. The radical scavenging activities are expressed in μmol Trolox/g of fresh fruit weight, results were 29.0 for DPPH and 39.5 for ABTS. Sample concentration was 0.1 g/mL.	Total flavonoids, total anthocyanins, gallic acid, catechin, ellagic acid, cyanidin-3-glucoside and cyanidin-3-rutinoside	[42]
Leave extracts, seed extracts and pulp extracts from raspberries	Chemical: ABTS, FRAP and DPPH	Leave extracts exhibited the strongest antioxidant activity, followed by pulp extracts and seed extracts. Sample concentrations were 30 μg/mL. DPPH and ABTS results were expressed as inhibition percentage; FRAP is expressed as in mM ferrous sulfate equivalents/g sample in dry weight, results are the following: leave extracts (ABTS ≈ 88%, DPPH ≈ 78%, FRAP ≈ 1105), pulp extracts (ABTS ≈ 76%, DPPH ≈ 73%, FRAP ≈ 1025) and seed extracts (ABTS ≈ 80%, DPPH ≈ 49%, FRAP ≈ 325).	Total phenols, avicularin, gallic acid, epicatechin, ellagic acid pentoside, ellagic acid, quercetin 3-*O*-glucoside, kaempferol-7-*O*-glucoronide, quercentin-7-*O*-glucuronide, rutin, procyanidin B2 and procyanidin C3	[88]
Raspberry pomace	Chemical: ORAC and ABTS	Raspberry pomace extracts were performed with methanol and hexane. For ABTS assay results with methanol were higher (308–561 mol TE/g) compared with hexane extracts (48.5–122.7 mol TE/g). Also, lipophilic fractions of raspberry pomace were isolated by supercritical carbon dioxide extraction, their ABTS capacity = 123.3 mol TE/g; ORAC = 936.2 mol TE/g.	Total phenolics, anthocyanins and ellagitannins	[85]
Raspberries extracted in 95% ethanol to obtain a powder	Chemical: FRAP In vitro: ABTS and DPPH	Seven different extracts from berries and fruits, including raspberry, were used at the same conditions. Raspberries and blackberries had the highest antioxidant capacity compared to the other fruits. FRAP activity = 103.9 ± 0.9 µM Fe^2+^/g. The highest antioxidant capacity results in vitro were obtained with the DPPH assay. ABTS inhibition percentage = 31.1 ± 0.6%. DPPH inhibition percentage = 87 ± 1.2%.	Total phenolics, flavonoids and proanthocyanidins	[89]
Raspberry leaf extracts	Chemical: FRAP, DPPH and ABTS	Average results by means of the DPPH assay were 70% higher compared to ABTS assay. Results were the following: FRAP = 20.77 ± 1.92 mM Fe(II), ABTS = 4.00 ±0.89 mM Trolox and DPPH = 6.84 ± 0.22 mM Trolox.	Total phenols, total flavonoids, total tannins, caffeic acid, ellagic acid, flavan-3-ols, hydroxycinnamic acid, quercetin, chlorogenic acid flavonols, flavones and isoflavones	[90]
Raspberry leaves from organic and conventional cultivars	In vitro: ABTS	Organic raspberry leaves had 26% higher antioxidant activity compared to the conventional raspberry leaves. Organic leaves ABTS capacity was 77.93 mmol Trolox/100 g FW, while for conventional leaves was 61.78 mmol Trolox/100 g of fresh weight. Concentrations of the samples used were 10 mg/mL.	Total phenols, total flavonoids, *p*-coumaric acid, quercetin, ellagic acid, chlorogenic acid, caffeic acid, luteolin, salicylic acid, myricetin, quercetin-3-*O*-glucoside and quercetin-3-*O*-rutinoside	[35]
Raspberry jam	In vitro: ABTS	Five different berry jams, including raspberry jam, were used at the same conditions. Raspberry and blueberry jams presented a lower antioxidant activity compared to blackcurrant, blackberry and cranberry jams. The ABTS capacity reported was 10.10 μM Trolox/g fresh of weight.	Total phenols, total flavonoids, cyanidin-3-*O*-sophoroside and cyanidin-3-*O*-sophoroside-rhamnoside	[86]

## 5. Anti-Inflammatory Activity

The inflammation response is tightly regulated by the immune system and is composed of multiple signaling steps. In the presence of tissue damage and/or pathogen infection, a downstream signaling cascade, which causes inflammation, is activated. Briefly, pathogen-associated molecular patterns (PAMPs) and damage-associated molecular patterns (DAMPs) recognize and bind to Toll-like receptors (TLRs), specific receptors located in the cytoplasm, the endosome and plasma membrane [91,92] and telencephalin (TLN) cells that compose the immune system. Once this recognition occurs, the stimulation source determines the duration and type of inflammatory response, which, in normal conditions, is only temporary. Although, in chronic diseases, inflammasome is activated for long periods, this continuous effect plays an important role in the development of cancer [93,94]. An inflammasome is a cytosolic multiprotein complex that allows the activation of proinflammatory caspases, leading to an inflammatory response, when pro-IL-1β is transformed into interleukin-1β (IL-1β). The inflammasome assembles by binding NLRP3 with ASC (apoptosis-associated speck-like protein) and inactive caspase-1. Analogous to this assembly, a ligand binds to the TLR, activating its TIR (Toll-interleukin receptor) domain, and then turns on a signal to another protein to initiate a response pathway that results in the presence of pro-IL-1β. When the inflammasome assembles, caspase-1 can convert pro-IL-1β to IL-1β, which is later released and secreted to ignite acute inflammation [95]. Multiple DAMPs are linked to cancer, causing a chronic inflammation in approximately 25% of cases. TLR-DAMP binding activates protein kinase signaling pathways, and this produces proinflammatory transcriptional factors such as NF-κB and interferon gamma. Moreover, NF-κB activation is widely known to promote tumorigenesis [96,97]. Other growth factors and receptors implicated in cancer initiation and progression are tumor necrosis factor-γ and inflammation-related cytokines IL 1, -2, -6 and -8 [51]. Additionally, inflammation can produce ROS and RNS, which affect the macromolecules and DNA. In cancer, there is evidence of a dysfunction in the antioxidative proteins by the ROS and RNS that results in higher oxidative stress [24].

Anthocyanins and gallic acid released by ellagitannins, when metabolized, have anti-inflammatory properties [59,73]. Multiple enzymatic systems are involved in the inflammation signaling pathways, particularly flavonoids, since they can affect the tyrosine and serine-threonine protein kinase signaling pathways [98]. Quercetin, catechin, caffeic acid, sinapic acid, ferulic acid and chlorogenic acid, which are found in raspberries, can inhibit the peroxidation of lipids [99]. Likewise, anthocyanins can reduce the expression levels of inducible nitric oxide synthase, cyclooxygenase-2, IL-1β and IL-6 [100], reducing inflammation. The anti-inflammatory activity of phenolic compounds may be due to their ability to interfere with oxidative stress signaling and suppress proinflammatory signaling transduction [81]. These compounds have been reported to block the secretion of proinflammatory cytokines [101]. Table 2 depicts research were the anti-inflammatory properties attributed to raspberries have been evaluated through in vitro and in vivo assays. The research by Mah et al. used the protein denaturation assay and nitric oxide (NO) assay to evaluate the anti-inflammatory properties of plant extracts; it used the Griess reagent to measure the inhibition of NO [102]. RAW 264.7 cells are one of the most reported cell lines used for anti-inflammatory capacity assays. The RAW 264.7 cell line is obtained from mouse monocyte macrophages; the male mouse is induced a tumor by the intraperitoneal injection of Abselon Leukemia Virus (A-MuLV) [103]. As described in Table 2, raspberry extracts can reduce the levels of interleukins and cyclooxygenases involved in inflammation signaling [100,104]. In particular, anthocyanins can reduce cyclooxygenase-2 expression due to the *ortho*-dihydroxyphenyl structure on their B-ring [105]. An inhibition of the transcriptional factors, such as NF-κB, which is involved in the tumor development by promoting cancer cell proliferation, has been reported [100,106,107,108]. Therefore, the anti-inflammatory effect of phenolic compounds assists in showing the anticancer activity.

## 6. Cytotoxicity

Cytotoxicity assays are used to determine the viability of cells after being exposed to a compound or substance. The number of live and/or dead cells is counted after the culture has an incubation period, and a control is required to correctly compare the effects of the compound being tested for the experiment [110]. Having a control as a way to validate the treatment is selective; this means it only attacks damaged cells, while healthy cells remain alive. Cytotoxicity assays have been performed in vitro on raspberry-extracted phenolic compound family dose limits to elucidate the concentrations at which cell death from direct contact or due to the leaching of toxic substances can be induced [111]. According to the end point measurements, the cytotoxicity assays can be classified into four categories: dye exclusion assays, colorimetric assays, fluorometric assays and luminometric assays. Colorimetric assays, such as MTT (3-(4,5-dimethylthiazol-2-yl)-2–5-diphenyltetrazolium bromide) and MTS (5-(3-carboxymethoxyphenyl)-2-(4,5-dimethyl-thiazoly)-3-(4-sulfophenyl) tetrazolium, inner salt), are commonly used due to their high reproducibility, accuracy and low costs compared to others. They are based on the evaluation of the metabolic activity in cells by measuring a biochemical marker [112]. Particularly, MTT is a DNA binding dye that should not be used for a long-term real-time detection of the accumulation of apoptotic cells due to its high toxicity after a few hours of exposure to the study cell lines [110].

The anticancer effect attributed to phenolic compounds found in raspberries is associated with multiple pathways, such as cell proliferation, invasion, autophagy and apoptosis. In the following studies, the cancer cytotoxic-specific capacities of the phenolic compounds, also found in raspberries, are described. Anthocyanins can inhibit the cell viability by downregulating the expression of PI3K/Akt and decreasing Bcl2/Bax [113]. Kaempferol induces autophagy in gastric cancer cells via the IRE1-JNK-CHOP pathway and inhibits the expression of the vascular endothelial growth factor [114]. Ferulic acid induces arrest in the G0/G1 phase in HeLa and CaSki cervical carcinoma cells [115]. Additionally, it reduces the expression of cyclin D1 and cyclin E and induces the expression of p53 and p21 [115,116]. The anticancer effect from gallic acid is related to its antitelomerase activity [117]. Ellagic acid has been proven to activate AMPK and inhibit HIF-1α in lung cancer [118]. In other studies, ellagic acid reduced the IL-6 expression gene in prostate cancer cells [119]. Overall, multiple authors have concluded that the anticancer effect attributed to phenolic compounds is dose-dependent. Most of raspberry phenolic compound studies done to evaluate their cytotoxicity were in vitro, as shown in Table 3. Each author used different dosages to determine their cytotoxicity, although most of them reported in their results no cytotoxicity. The results are commonly expressed by cell viability inhibition (IC_50_) and growth inhibition (IG_50_), depending on the experiment conditions and assay used. Moreover, it is important to determine the LD_50_ and maximum dosage tolerated by the live cells. The studies from Table 3 summarize the raspberry extract dosages in which different cell lines were exposed to determine their effect on cell viability. Particularly, in cancer cell lines, a low cell viability is a positive finding. The in vitro and in vivo studies showed a growth inhibition in cancer cells when raspberry extracts were applied to them.

## 7. Conclusions

Raspberries have been proven, by multiple sources, to have a high antioxidant and anti-inflammatory activity that patients with chronic diseases may benefit from. Intestinal microbiota plays an important role in the metabolism and absorption of phenolic compounds and their derivatives. The possible modulatory effect between bacteria and phenolics is an interesting study approach to evaluate when studying the human health benefits attributed to raspberries. The concentration of beneficial gut bacteria is indispensable for a higher absorption of phenolic compounds. Therefore, the antioxidant and anti-inflammatory effects attributed to raspberries will vary between humans. Most research studies on raspberries have focused on the total phenolic fractions, such as anthocyanins or ellagitannins, rather than specific compounds. This field of study has a wide opportunity for new research works due to the numerous quantities of phenolic compounds found in nature. For future research, we recommend describing the cultivar conditions of the samples, since a variation of the physicochemical characteristics between the same species has been reported due to this reason. Moreover, it is important to elucidate accurate information regarding the concentrations of these compounds in raspberries. Health-aiding activities may vary, depending on multiple factors, such as the assays used for evaluation, since each approach is different, and not all are applicable to both lipophilic and hydrophilic antioxidants; others are based on the location of the matrix obtention, extraction methods and applied treatments. Additionally, analyzing raspberry genotypes from different harvest seasons and regions is relevant due to the variability observed in previous studies. Regarding an antioxidant activity evaluation, it is recommended to perform at least two different assays, because each one is based on a specific oxidative reaction, for the purpose of reporting more accurate results. Therefore, some compounds may present a higher affinity for an assay due to their chemical structure. Raspberry phenolic family compounds act as effective antioxidants when pairing the free electron with reactive oxygen species (ROS). Extensively studied sources used raspberry extracts to reduce inflammation in in vitro and in vivo scenarios, considering this is one of the main effects that promote the development of cancer, mainly by DAMPs. Phenolic compounds can inhibit the signaling pathways for NF-κB and MAPK expression in the inflammatory response. Abnormal expression of the inflammatory factors plays a critical role in cancer development. Therefore, the potential co-adjuvant effect raspberry extracts have against inflammation is an interesting finding. Comparing studies that have both in vivo and in vitro models is important to check if the results are accurate enough. Both inflammatory and antioxidant effects have correlations between them, because in chronic diseases, both patients are present. Finally, the phenolic compounds cytotoxicity assay is a key point in determining the maximum tolerated dosage and preventing cell death. In previously mentioned studies, the cell viability was reduced in cancer cell lines. In conclusion, the raspberry phenolic compound family might be a potential co-adjuvant for the reduction of oxidative stress and inflammation in chronically diseased patients and as a prevention care treatment. Further research to examine the antioxidant and anti-inflammatory effects will provide new information on which specific compounds, from raspberries, exhibit better health benefits with the lowest cytotoxicity.

## Figures and Tables

**Table 2:** Studies performed in the in vitro and in vivo assays to determine the anti-inflammatory activity when using the extracts or fractions from raspberries (*Rubus idaeus*).

Model	Source	Results	Reference
**In vitro**			
LPS-stimulated microglia	Raspberry extract enriched in ellagitannins and ellagic acid derivatives Dose: 1 µg GAE/mL	Increased the release of the anti-inflammatory cytokine IL-10. Attenuated pro-inflammatory markers and mediators CD40, NO, TNF-α, and intracellular superoxide via NF-κB, MAPK and NFAT pathways.	[108]
LPS/IFN-γ-activated RAW 264.7 macrophages	Crude extracts, anthocyanin-rich fractions, and des-anthocyanin fractions from dried raspberry Dose: 200 mg/mL	The best anti-inflammatory results were obtained with the anthocyanin-rich fractions: highest efficiency in suppressing nitric oxide synthesis, reduced the levels COX-2, IL-1β and IL-6; and inhibited the activation of NF-κB and MAPKs. Decreased the phosphorylation of IKK, IκBα, p65 and JNK and the nuclear translocation of p65.	[100]
**In vivo**			
Dextran sulfate sodium– induced chronic colitis in mice	Dried raspberry Dose: 5% of dry feed weight	Mice who received dried raspberry supplementation in their diet, showed: facilitated epithelium repair, a reduced expression of inflammatory mediators (by 20–70%; *p* ≤ 0.01), infiltration of CD4 T cells (by 50%; *p* ≤ 0.05), and α4β7 integrin and related adhesion molecules (by 33.3%; *p* ≤ 0.01). Enhanced p53 stability by 53% and oncogenic gene expression was reduced by 50–60%.	[109]
Dextran sulfate sodium– induced chronic colitis in mice	Crude extracts, anthocyanin-rich fractions, and des-anthocyanin fractions from dried raspberry Dose: 20 mg/kg	Anthocyanin-rich fractions reduced DSS-induced acute colitis by inhibiting the activation of NF-κB and MAPKs, producing lower levels of inflammation and a reduction in morphological alteration. DSS-induced weight loss and histological damage were significantly ameliorated by ARFs treatment.	[100]
Mice with acute lung injury	Ellagic acid Dose: 10 mg/kg	Ellagic acid reduced IL-6 and COX-2 induced exacerbation of inflammation. Additionally, vascular permeability changes and neutrophil recruitment to the bronchoalveolar lavage fluid and to lung were reduced.	[104]

Abbreviations: dextran sulfate sodium (DSS), lipopolysaccharide (LPS), gallic acid equivalent (GAE), interleukin-1 beta (IL-1β), interleukin (IL-6), interleukin-10 (IL-10), cyclooxygenase-2 (COX-2), mitogen-activated protein kinases (MAPKs) and nuclear factor kappa-light-chain-enhancer of activated B cells (NF-κB).

**Table 3:** In vitro and in vivo cytotoxicity assays performed for raspberry (*Rubus idaeus*) extracts.

Model	Source	Results	Reference
**In Vitro**			
RAW 264.7 cells	Crude extracts, anthocyanin-rich fractions, and des-anthocyanin fractions from dried raspberries	The MTS assay showed no significant changes in cell viability at dosage up to 200 μg/mL, they presented no cytotoxicity.	[99]
Lung cancer A-549 cells	Raspberry seed extracts	When performing an MTT assay at as dosage up to 30 µg/mL; it was inhibited only the growth of the lung cancer A-549 cells (IC_50_ = 14.07 ± 0.96 µg/mL).	[119]
N9 murine microglial cells	Raspberry extract rich in ellagitannins and ellagic acid derivatives	MTT assay at a dosage of 1 µg GAE/mL and presented no cytotoxicity.	[107]
RAW 264.7 cells	Raspberry extract	At a dosage up to 200 μg/mL, the MTT assay showed no cytotoxicity.	[120]
RAW 264.7 cells	Anthocyanin fractions from raspberries	At a dosage up to 50 μg/mL, no cytotoxicity was observed with the MTT assay.	[120]
Human microvascular endothelial cells	Raspberry phenolic compound extract	The growth inhibition by 50% (GI_50_) was 87.64 ± 6.59 μg GAE/mL when performing an MTS assay.	[121]
Human colon adenocarcinoma cell line (Caco-2)	Raspberry ellagitannin preparation	At a concentration of 160 μg/mL, the cytotoxicity was higher than 70% in MTT and 80% in the PrestoBlue assay.	[122]
Human leukemia cell lune: J45 and HL60	Raspberry juice extract	Cell viability inhibition by 50% (EC_50_) was 0.1875 mg FW/mL for the HL60 line and 0.0815 mg/mL for the J45 line when performing the trypan blue staining method.	[40]
Human laryngeal carcinoma (HEp2) and human adenocarcinoma of the colon (SW 480)	Dried raspberry leaf extracts	For the neutral red assay, a concentration of 0.01g/mL was used. Cytotoxicity was higher in SW 480 than HEp2 cells after 24 h of exposure. At 1 h of exposure, no toxic effect was seen in both cell lines. The cell viability inhibition by 50% reported for SW 480 = 3.25 g/100 mL and HEp2 = 2.34 g/100 mL.	[89]
**In Vivo**			
Mouse colitis model	Anthocyanin-rich fractions from dried raspberries	No cytotoxicity was reported at a 200 μg/mL concentration.	[99]
Kunming mouse model. B16F10 melanoma cells for tumor implantation	Raspberry pulp polysaccharides	At a dose of 400 mg/kg, a 59.95% growth inhibition ratio of melanoma was observed. No cytotoxicity was observed at a raspberry pulp polysaccharides concentration of 2000 mg/kg. Therefore, the LD_50_ was determined to be more than this concentration.	[123]
NMRI mouse model implanted with EAC (Ehrlich-Lettre ascites carcinoma) tumor cells	Raspberry pomace extract	Extracts showed cytotoxic properties against EAC cells. A dose of 2.0 mg/kg has a cancer-preventing activity.	[124]

Abbreviations: gallic acid equivalent (GAE), half-maximal inhibitory concentration (IC_50_), cell viability inhibition by 50% (EC_50_), growth inhibition by 50% (GI_50_) and lethal dose by 50% (LD_50_).
